# Therapeutic Effect of Benidipine on Medication-Related Osteonecrosis of the Jaw

**DOI:** 10.3390/ph15081020

**Published:** 2022-08-18

**Authors:** Ken Matsunaka, Mikio Imai, Koma Sanda, Noriyuki Yasunami, Akihiro Furuhashi, Ikiru Atsuta, Hiroko Wada, Yasunori Ayukawa

**Affiliations:** 1 Section of Implant and Rehabilitative Dentistry, Division of Oral Rehabilitation, Faculty of Dental Science, Kyushu University, Fukuoka 812-8582, Japan; 2 Division of Advanced Dental Devices and Therapeutics, Faculty of Dental Science, Kyushu University, Fukuoka 812-8582, Japan; 3 Laboratory of Oral Pathology, Division of Maxillofacial Diagnostic and Surgical Sciences, Faculty of Dental Science, Kyushu University, Fukuoka 812-8582, Japan

**Keywords:** benidipine, medication-related osteonecrosis of the jaw, TNF-α, jaw diseases, bisphosphonate

## Abstract

Medication-related osteonecrosis of the jaw (MRONJ) is an intractable disease that is typically observed in patients with osteoporosis or tumors that have been treated with either bisphosphonate (BP) or antiangiogenic medicine. The mechanism of MRONJ pathogenesis remains unclear, and no effective definitive treatment modalities have been reported to date. Previous reports have indicated that a single injection of benidipine, an antihypertensive calcium channel blocker, in the vicinity of a tooth extraction socket promotes wound healing in healthy rats. The present study was conducted to elucidate the possibility of using benidipine to promote the healing of MRONJ-like lesions. In this study, benidipine was administered near the site of MRONJ symptom onset in a model rat, which was then sacrificed two weeks after benidipine injection, and analyzed using histological sections and CT images. The analysis showed that in the benidipine groups, necrotic bone was reduced, and soft tissue continuity was recovered. Furthermore, the distance between epithelial edges, length of necrotic bone exposed in the oral cavity, necrotic bone area, and necrotic bone ratio were significantly smaller in the benidipine group. These results suggest that a single injection of benidipine in the vicinity of MRONJ-like lesions can promote osteonecrotic extraction socket healing.

## 1. Introduction

Medication-related osteonecrosis of the jaw (MRONJ) is a rare and intractable disease that was first reported in 2003 [1]. MRONJ was first reported among recipients of bisphosphonate (BP); then, patients who were administered an antireceptor activator of nuclear factor κB ligand (denosumab) [2] or antiangiogenic medicine [3] were also reported to have similar symptoms [4]. Few curative remedies have been reported for MRONJ, but palliative treatments have been proposed, such as irrigation [5], antibiotic medication [6], laser therapy, and platelet-rich plasma therapy [7] with the removal of bone sequestrum [8]. However, sequestrectomy results in re-exposure of bone in the oral cavity and an increased chance of subsequent osteonecrosis recurrence. In addition, en bloc bone sequestration increases the difficulty of occlusal reconstruction with dentures, which makes it difficult to obtain stability, support, and retention on a flat or concave alveolar ridge. Parathormone treatment has been proposed as a curative treatment modality for MRONJ [6]. However, the modality of this potential treatment has a large drawback in that hormonal agents such as parathormone are unsuitable for patients with cancer [9]. In particular, since BP and denosumab are often used to prevent bone metastasis of tumor cells, and antiangiogenic drugs are a type of anticancer drug, the number of patients for which parathormone can be used as an anti-MRONJ agent is relatively low in light of expectations.

The mechanism of MRONJ onset still remains to be elucidated; however, the function of BP and other drugs that suppress neovascularization [10], suppress bone remodeling [11], or increase the chance of bacterial infection in the oral cavity [12] may have a severe impact on MRONJ development.

MRONJ is a disease that is characterized by the infection of bone by oral bacteria. A hypothesis can be created in that accelerated gingival wound healing may reduce the chance of bone exposure in the oral cavity and subsequent bone infection. In our recent report, benidipine (BD), a calcium channel blocker that has been used as an antihypertensive and antiarrhythmic agent [13,14,15], was found to promote the healing of gingival soft tissue around tooth extraction sockets [16]; thus, we hypothesized that BD is a possible candidate for a curative remedy. In addition, some culturing studies indicate that BD can enhance bone formation [17,18,19,20]. Our previous report also indicated that BD can accelerate bone healing in tooth extraction sockets [16]. These studies evoke the possibility that BD has modulatory functions on bone formation, which may also play a crucial role in the onset and/or healing of MRONJ.

In the present study, we verified the possibility of BD as a therapeutic agent in the healing of MRONJ. The definition of MRONJ is strict [4], namely: 1. current or previous treatment with antiresorptive therapy alone or in combination with immune modulators or antiangiogenic medications, 2. exposed bone or bone that can be probed through an intraoral or extraoral fistula(e) in the maxillofacial region that has persisted for more than eight weeks, and 3. no history of radiation therapy to the jaws or metastatic disease to the jaws. We previously established an MRONJ-like lesion model using rats [21], which followed the definition of MRONJ, excluding the bone exposure period, and we employed it in the present research. The null hypothesis here was that BD is not effective on MRONJ-like lesions in rats. To investigate this hypothesis, we administered a single injection of BD in the vicinity of MRONJ-like sites in tooth extraction sockets in rats and observed the change in the necrosed extraction socket using histology, histomorphometry, and μCT analyses.

## 2. Results

**Figure 1:**
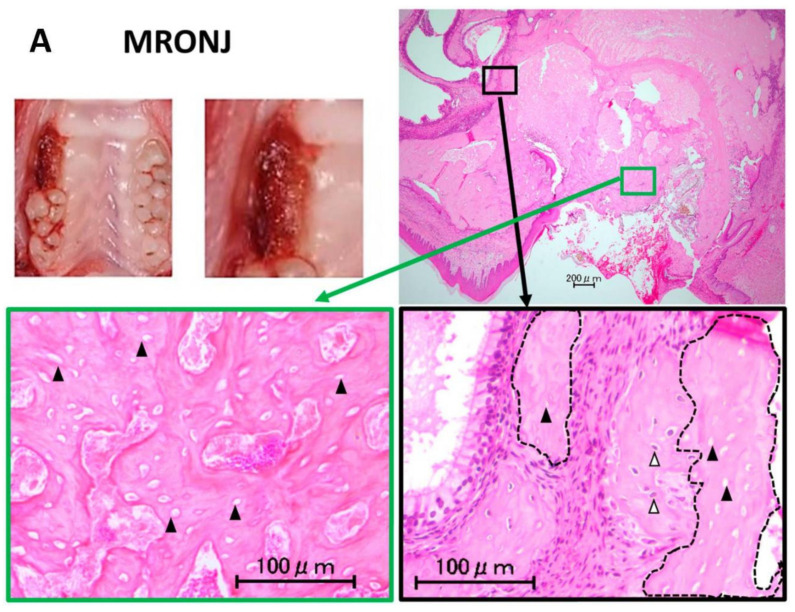

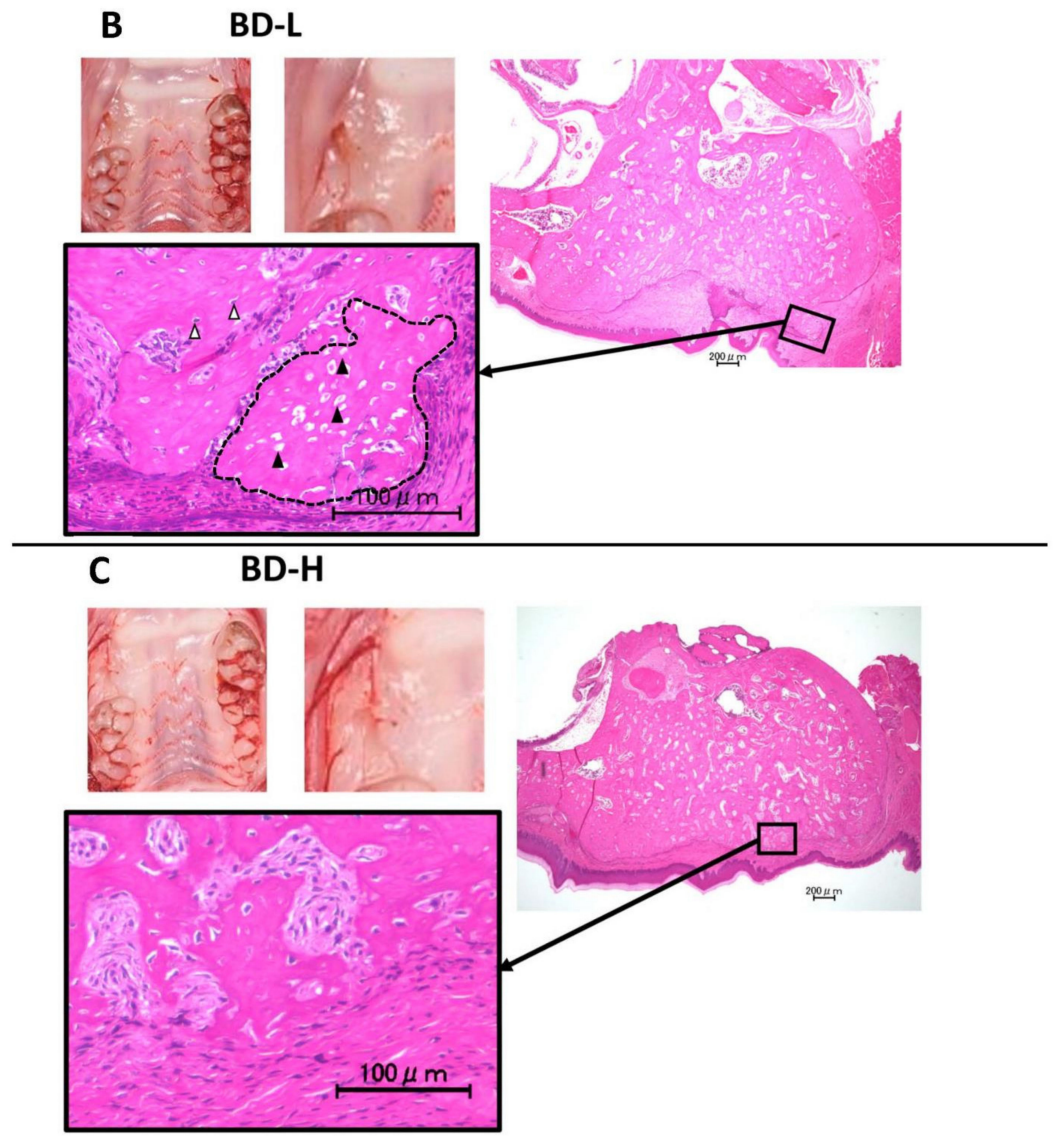
Intraoral and histological findings four weeks after extraction of the upper right first molar. (**A**). In the MRONJ group, epithelial continuity is disrupted, and necrotic bone characterized by vacant osteocytic lacunae (black arrowheads) is exposed at the coronal region (green square). At the periapical legion, both necrotic bone (the area circled by dotted lines) and vital bone with living osteocytes (white arrowheads) are observed (black square). (**B**). In the BD-L group, epithelial continuity is restored, and new bone formation is observed. Although small amount of necrotic bone (the area circled by dotted lines) characterized by vacant osteocytic lacunae (black arrowheads) is observed in the coronal region, to a great extent extraction socket is occupied by vital bone with living osteocytes (white arrowheads). (**C**). In the BD-H group, epithelial continuity is restored, and new bone formation is observed. Vital bone with living osteocytes is observed even at the coronal region of the extraction socket.

**Figure 2:**
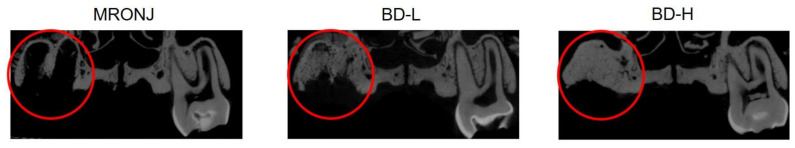
μCT findings in the center of the extraction sockets. More new bone formation is observed in the BD-administered group than the MRONJ group.

The efficacy of BD treatment on MRONJ-like symptoms was examined. In the MRONJ group, the histological sections showed that, in all rats, the extraction socket had broken epithelial continuity, necrotic bone was exposed, connective tissue formation was inadequate, and inflammatory cells were abundant around the necrotic bone (Figure 1A). Necrotic bone was common in the exposed area, but not around the root apex (Figure 1A). However, no MRONJ-like symptoms were seen on the contralateral (non-extraction) side. μCT examination revealed that little new bone was formed in the extraction socket of the MRONJ group, and the morphology of the extraction socket was nearly unchanged (Figure 2). Some histological sections in the BD low-dose group showed a restored or completely sealed epithelium (Figure 1B). Connective tissue formation was also seen under the epithelium with restored continuity (Figure 1B). In addition, there was a decrease in necrotic bone and inflammatory cells compared with the MRONJ group. The μCT examination results showed the formation of new bone within the extraction socket (Figure 2). In addition, the histological section findings in the BD high-dose group showed a restored or completely sealed epithelium in some cases, similar to the BD low-dose group (Figure 1C). The results of the μCT examination showed the formation of new bone within the extraction socket (Figure 2).

**Figure 3:**
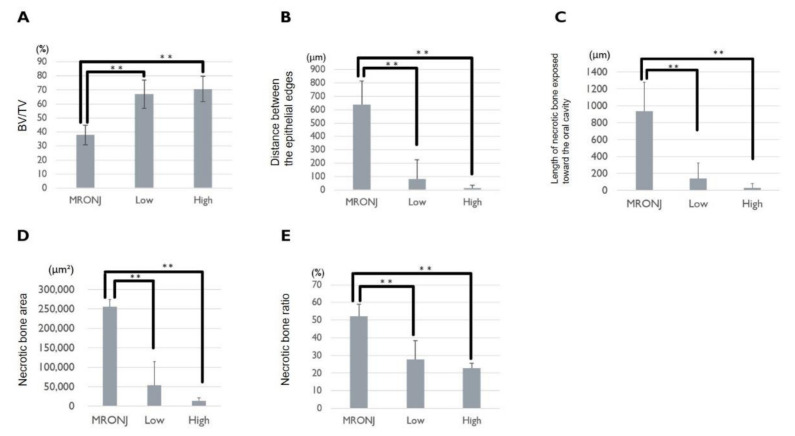
Five variables for evaluating the therapeutic effect of BD on MRONJ-like lesions. In the MRONJ, low, and high groups, the (**A**) BV/TV, (**B**) distance between the epithelial edges, (**C**) length of necrotic bone exposed toward the oral cavity, (**D**) necrotic bone area, and (**E**) necrotic bone ratio are measured, and statistical analyses are performed (Tukey’s test; **: *p* < 0.01).

Statistical analysis showed that BV/TV was significantly greater in the BD low-dose and BD high-dose groups than in the MRONJ group (Tukey’s test, MRONJ vs. BD-low dose: *p* < 0.01; MRONJ vs. BD high-dose: *p* < 0.01) (Figure 3A). The distance between the epithelial edges was significantly shorter in the BD low-dose and BD high-dose groups than in the MRONJ group (Tukey’s test, MRONJ vs. BD low-dose: *p* < 0.01; MRONJ vs. BD high-dose: *p* < 0.01) (Figure 3B). The length of necrotic bone exposed toward the oral cavity was significantly shorter in the BD low-dose and BD high-dose groups than in the MRONJ group (Tukey’s test, MRONJ vs. BD low-dose: *p* < 0.01; MRONJ vs. BD high-dose: *p* < 0.01) (Figure 3C). The necrotic bone area was significantly smaller in the BD low-dose and BD high-dose groups than in the MRONJ group (Tukey’s test, MRONJ vs. BD low-dose: *p* < 0.01; MRONJ vs. BD high-dose: *p* < 0.01) (Figure 3D). The necrotic bone ratio was smaller in the BD low-dose and BD high-dose groups than in the MRONJ group (Tukey’s test, MRONJ vs. BD low-dose: *p* < 0.01; MRONJ vs. BD high-dose: *p* < 0.01) (Figure 3E).

## 3. Discussion

In our previous study using healthy rats, topical and single injections of BD promoted soft- and hard-tissue healing around tooth extraction sockets through enhancement of the migration and proliferation of gingival epithelial cells and fibroblasts [16]. From the viewpoint of the effect of BD on bone metabolism, this drug reportedly enhances osteoblastic alkaline phosphatase activity, enhances mineral matrix deposition, and decreases receptor activation of nuclear factor kappa-B ligand expression [20]. Other reports regarding the activity of BD in osteoblasts include upregulation of the gene expression levels of runt-related transcription factor-2, bone morphogenetic protein-2, and osteocalcin [19]. In addition, there is blocking of the T-type Ca channel, which is reported to deactivate Rho kinase [22], and a Rho kinase inhibitor, Y-27632, reportedly increases BMP-4 production [23]. These collectively enhance the differentiation of bone marrow stromal cells into osteoblasts [24]. Thus, we assumed that BD could enhance both the soft- and hard-tissue healing of tooth extraction sockets, even with medication-induced bone necrosis, as well as of healthy extraction sockets. In the present study, more bone formation was found in the BD groups, and the area and ratio of necrotic bone was observed to decrease in the BD groups. As a possible indirect effect of BD on MRONJ healing, several Ca channel blockers, including BD, are reported to promote neovascularization and anti-inflammation [25]. Our results show the effect of BD in reducing both the area and ratio of necrotic bone. The neovascularizing activity of BD may contribute to reducing the necrotic bone area. In addition, BD and other L-type Ca channel blockers are known to block the production and function of tumor necrosis factor (TNF)-α and the subsequent inflammatory reaction [25]. The reduction in TNF-α synthesis caused by BD is another benefit that may be expected from MRONJ treatment. TNF-α is known to facilitate monocyte differentiation into M1 macrophages [26]. M1 macrophages are known to induce persistent inflammation [27]. In fact, a previous report indicated that BP administration increases the number of M1 macrophages [28]. In the present study, in addition to its effects on suppressing inflammation, it is possible that BD attenuates the BP-induced increase in the number of M1 macrophages.

In the present study, epithelial continuity showed higher recovery in the BD groups. Gingival overgrowth, as a result of fibroblastic collagen overproduction, is a side effect associated with some Ca channel blockers [29]. However, since this side effect has not been reported for BD [29], the earlier gingival healing induced by BD in the present study cannot be ascribed to gingival overgrowth. In a previous report, T-type Ca channel blockers, including BD, were found to inhibit Rho kinase activity [22], as indicated above. Since the inhibition of Rho/Rho-associated kinase/myosin signaling is known to enhance cell migration [30], the blocking of this signaling by BD in the present study may promote soft-tissue cell migration at the site, with subsequent accelerated wound closure.

BD has been used as an oral medicine in the treatment of hypertension. However, the peroral administration of BD may have less of an impact on MRONJ because BD is reported to have low systemic bioavailability with the characteristics of an immediate increase in plasma concentration and bi-exponential declination [31], which is why we chose topical injections as the administration route. Moreover, it can be expected to reduce systemic influences, such as the unexpected pressure drop caused by the oral administration of BD.

This study had some limitations. First, as described above, since we could not strictly fulfill the criteria of MRONJ in our model, we tested the effect of BD on a rat model that exhibited MRONJ-like symptoms. Next, the animal in the present study does not entirely reflect clinical conditions because MRONJ is typically seen in elderly patients, but in the present study, elderly rats were not employed due to their unavailability. In addition, the optimal dose, number of administrations, and administration period were not tested.

## 4. Materials and Methods

### 4.1. Animals

A total of 21 Wistar rats (female, 4 weeks old, 55–70 g body weight, in 3 groups of 7) were used. All the experiments were performed according to the ARRIVE guidelines for reporting animal research. All procedures for this experiment were approved by the Institutional Animal Care and Use Committee of Kyushu University (approval number: A21-402-0) and are in accordance with the Guide for the Care and Use of Laboratory Animals (7th and 8th editions, ILAR-NRC).

### 4.2. MRONJ-like Rat Model

An MRONJ-like rat model was previously established [21] based on the report by Kaibuchi et al. [32]. The rats in all groups were injected with 66 μg/kg of zoledronic acid (BP; Zometa; Novartis Pharma, Tokyo, Japan) and 5 mg/kg of dexamethasone (Dex; Decadron; Aspen Japan, Tokyo, Japan) subcutaneously three times a week. Two weeks after the start of BP and Dex administration, 0.15 mg/kg of medetomidine hydrochloride (Medetomin; Meiji Seika Pharma, Tokyo, Japan), 2 mg/kg of midazolam (Dormicum; Maruishi Pharmaceutical, Osaka, Japan), and 2.5 mg/kg of butorphanol tartrate (Vetorphale; Meiji Seika Pharma, Osaka, Japan) were administered intraperitoneally, and the right maxillary first molar was extracted under general anesthesia. Bone exposure was confirmed two weeks after tooth extraction. Four weeks after tooth extraction, the rats were euthanized via injection with 12 mg/kg of pentobarbital sodium (Somnopentyl; Kyoritsu Seiyaku, Tokyo, Japan).

### 4.3. Application of BD

**Figure 4:**
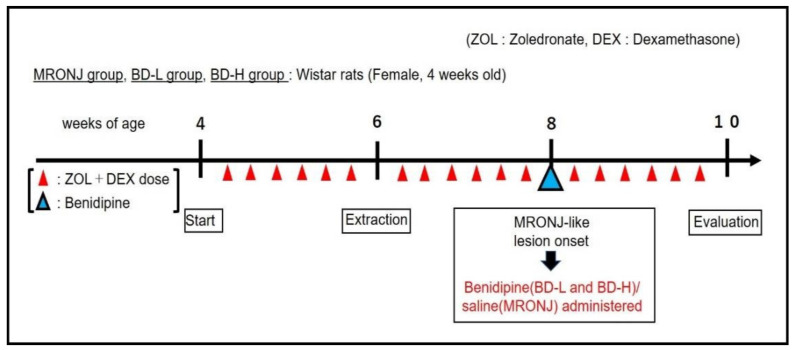
Experiment timeline.

A single dose of benidipine (BD; Wako Pure Chemical Industries, Osaka, Japan) was administered near the site of MRONJ symptom onset in a model rat that developed MRONJ-like symptoms 2 weeks after tooth extraction (Figure 4). Two BD concentrations, a low dose (0.13 mg/kg; BD low-dose group, *n* = 7) and a high dose (1.3 mg/kg; BD high-dose group, *n* = 7), were suspended in saline and injected (27 G × 3/4; TERUMO, Tokyo, Japan) at 0.1 mL/100 g rat. The MRONJ group received the same volume of saline at the same site (*n* = 7). Rats were randomly assigned to each group. Four weeks after tooth extraction, the animals were euthanized by injection with 12 mg/100 g of sodium pentobarbital (Somnopentyl; Kyoritsu Seiyaku, Tokyo, Japan). A comparison of the BD-treated and MRONJ groups was made based on μCT and histological section findings.

### 4.4. μCT Examination and Morphometry

After rat slaughter, maxillary bones were harvested and fixed with 10% paraformaldehyde (Merck, Darmstadt, Germany) for 24 h. A μCT scan was then performed (SkyScan 1076; Bruker microCT, Kontich, Belgium; tube current: 201 μA; voltage: 49 kV; pixel size: 9 μm). The bone volume (bone volume/tissue volume: BV/TV) in the extraction socket was measured using image analysis software (CTAn; Bruker microCT, Kontich, Belgium).

### 4.5. Histology and Histomorphometry

After scanning with μCT, the maxillary bones were demineralized with 20% ethylenediaminetetraacetic acid (Dojindo Laboratories, Kumamoto, Japan), dehydrated with alcohol (99% synthetic ethanol; Mitsubishi Chemical, Tokyo, Japan), permeabilized with xylene (Nacalai Tesque, Kyoto, Japan), and embedded in paraffin. Sections (3 μm thick) were sliced parallel to the frontal plane and stained with hematoxylin and eosin. The paraffin was dissolved in xylene, dehydrated with ethanol, and stained using a staining solution. After further dehydration with an ethanol solution series of ascending concentration and xylene, the sections were sealed with an encapsulant (Entellan^TM^ New for Microscopy; Merck). The sections were examined under an optical microscope (BZ-9000; Keyence, Osaka, Japan). Measurements were taken at the center of the extraction socket, 100 μm proximal to the center and 100 μm distal to the center. The evaluated parameters were the distance between the epithelial edges, length of necrotic bone exposed toward the oral cavity, necrotic bone area, and necrotic bone ratio. For the interepithelial distance, the shortest distance between the severed ends of the epithelium where continuity had been broken was measured. The interepithelial distance was set to 0 if the epithelial continuity was completely restored. For the distance of exposed necrotic bone, the longest distance of exposed necrotic bone was measured. For rats in which the epithelial continuity was completely restored, the necrotic bone exposure distance was set to 0. The extent of the necrotic bone area was measured, which was defined as the area of the bone with vacant osteocytic lacunae. The necrotic bone ratio was measured as the ratio of the vital bone to the necrotic bone within a square of 250,000 μm^2^ in the bone-exposed area. All measurements were taken three times, and the average was calculated.

### 4.6. Statistical Analyses

The Kolmogorov–Smirnov test was performed to confirm normality, and Tukey’s test was performed for comparison between multiple groups (3 groups), since the Bartlett test results showed equal variances. R (The R Foundation for Statistical Computing, Vienna, Austria) was used for all statistical analyses. *p* < 0.05 was considered to indicate a statistically significant difference.

## 5. Conclusions

A single and topical injection of BD adjacent to tooth extraction sockets in rats with MRONJ-like symptoms reduced the exposed and necrotic bone area and promoted new bone formation. These results suggest that the administration of BD in this manner is a potential novel MRONJ remedy.

## Data Availability

Data is contained within the article.
